# Perforated appendicitis due to fishbone

**DOI:** 10.1093/jscr/rjad694

**Published:** 2024-01-04

**Authors:** Van T Hoang, The H Hoang, Hoang Q Nguyen, Ngoc T T Pham, Tien H Vo, Vichit Chansomphou, Duc T Hoang

**Affiliations:** Department of Radiology, Thien Hanh Hospital, Buon Ma Thuot, Vietnam; Department of Radiology, Thien Hanh Hospital, Buon Ma Thuot, Vietnam; Department of Radiology, Family Hospital, Da Nang, Vietnam; The University of Danang - School of Medicine and Pharmacy, Da Nang, Vietnam; Department of Radiology, Tam Tri Nha Trang General Hospital, Nha Trang, Vietnam; Department of Radiology, Savannakhet Medical-Diagnostic Center, Kaysone Phomvihane, Laos; Division of Endocrinology, Department of Medicine, Walter Reed National Military Medical Center, Bethesda, United States

**Keywords:** appendicitis, computed tomography, fishbone, foreign body, perforation

## Abstract

Appendicitis is a common condition in daily clinical practice. Appendicitis due to foreign bodies is uncommon and may result from obstruction or perforation mechanism. We present a rare case of a 43-year-old male patient who was diagnosed with perforated appendicitis due to a fish bone by imaging studies and confirmed postoperatively. Confirming the fish bone causing the perforation on images is sometimes difficult, requiring the radiologist to actively search and determine the source. In addition to appendectomy, the surgeon also needs to pay attention to removing all foreign objects and treating perforations of surrounding organs.

## Introduction

Most foreign bodies tend to pass naturally through the digestive tract within a week or sometimes longer. A small number of them can cause gastrointestinal symptoms and complications due to swallowing of foreign bodies are ˂1% [1, 2]. Gastrointestinal perforation due to foreign body ingestion is very rare and is usually due to sharp foreign bodies. Intestinal injury due to foreign body ingestion tends to occur in areas with sharp angles but has been reported in all segments. Fish bones are the most common ingested object, most commonly causing perforation of the ileum, followed by the duodenal and colonic C-loop. Foreign bodies in the appendix are an uncommon finding with the incidence of all foreign bodies in the lumen of the appendix reported between 0.005% and 0.113% [3, 4]. Fishbone foreign bodies in the appendix are extremely rare with the potential for perforation of the appendix due to the reduced flexibility of the fishbone to accommodate changing intestinal motility patterns. The incidence of perforated appendicitis due to fish bones is unknown, estimated to be ˂0.0005% [3–5]. We report a rare case of perforated appendicitis due to fish bone, diagnosed by ultrasound and computed tomography (CT).

## Case presentation

**Figure 1:**
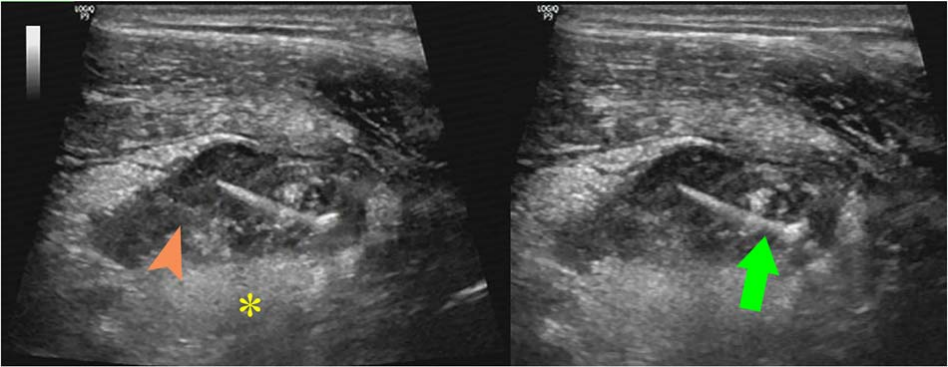
Ultrasound images showed a large pus-filled (arrowhead) appendix with a thin internal echogenic structure (arrow) extending outward from the muscle layer with surrounding fat infiltration (asterisk).

**Figure 2:**
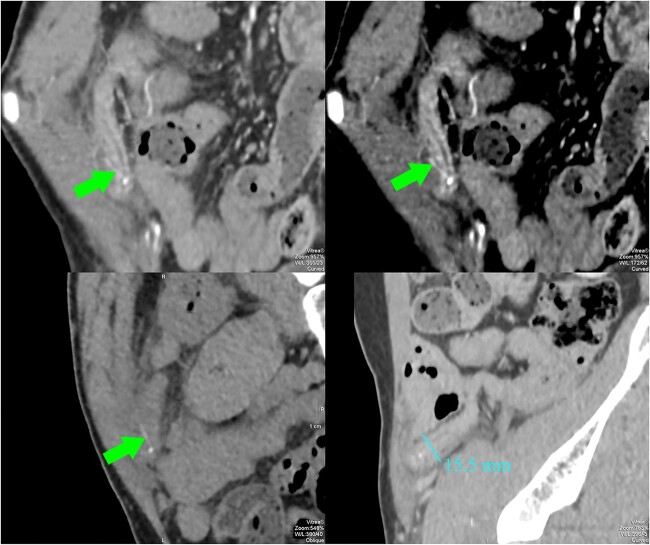
CT images showed a dilated appendix (maximum diameter 15.5 mm) with thickened and hyperenhancing wall. A small amount of free fluid and fat stranding was seen in the right iliac fossa. There was a linear hyperdensity measuring 13 mm (arrows) perforating and extending slightly beyond the appendix. No abscess or free gas collection was detected.

**Figure 3:**
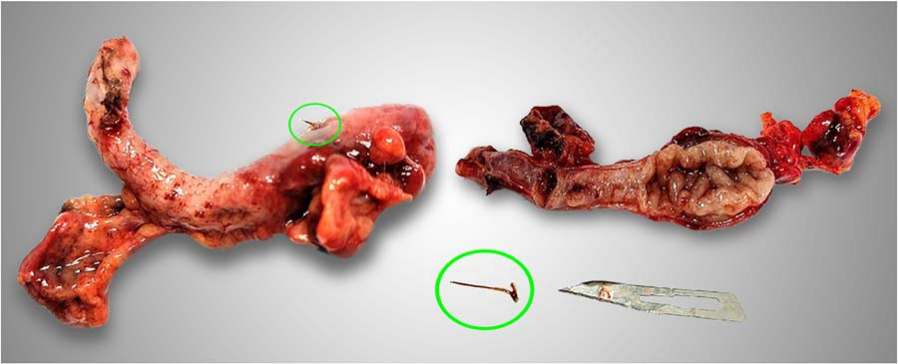
Photograph of the surgical specimen showed appendicitis due to fish bone (circles).

A 43-year-old man was hospitalized with dull pain in the right lower quadrant of the abdomen for 3 days. Accompanying symptoms were low-grade fever and nausea. In the emergency department, vital signs showed blood pressure 130/80 mmHg, heart rate 80 beats per minute, body temperature 37.9°C, and respiratory rate 22 per minute. Clinical examination revealed localized rebound tenderness on palpation at the right lower abdominal quadrant. Laboratory findings showed elevated white cell count of 15 000/mL (normal value <11 000/mL). Abdominal ultrasonography showed a large pus-filled appendix with an internal thin echogenic structure extending outward from the muscle layer and fatty infiltrates in the right iliac fossa (Fig. 1). In the ultrasound room, after being explained by the sonographer about the suspicion of appendicitis due to foreign bodies, the patient remembered and reported that he ate fish 5 days ago. CT scan performed then confirmed perforated appendicitis caused by a foreign body (Fig. 2). The patient underwent laparoscopic appendectomy without complications. The postoperative diagnosis was perforated appendicitis caused by fishbone (Fig. 3). The patient recovered and was discharged within 4 days.

## Discussion

Many foreign bodies have been reported in the literature as causes of acute appendicitis including metallic objects (bobby pins, lead shot, bullets, mercury liquid, earring, nails, endodontic file, needles, fish hook, pins, intrauterine contraceptive device, safety pins, jackstone/child’s game, screws, key, tacks, coins, drills, tongue rings), plant materials (fruit, seeds and pits, wooden splinters and toothpicks, thorns), animal or human materials (bones and portions of bone, hair, eggshell fragments, parasitic worms, gallstones, teeth and portions of teeth, dental crowns), miscellaneous (chewing gum, match fragment, condom fragment, paraffin, dental amalgam, plastic pieces, fishing line, stones, gambling dice, thermometer fragment, gastric tube tips, thread, toothbrush bristles, or birdshot) [4, 5].

The gravity-dependent location of the appendix makes foreign bodies tend to be attracted and deposited there. In addition, the ability to enter and exit the appendix lumen also depends on the size of the appendix hole (it can be closed or wide open, easy to enter and difficult to exit). Once the foreign body is in the appendix, peristaltic movements are often insufficient to expel it back into the cecal lumen. Usually objects with blunt edges will cause inflammation by blocking the lumen of the appendix, while sharp objects tend to cause inflammation by perforating the appendix wall, causing more serious complications such as appendiceal abscess, perforation of adjacent organs, and peritonitis [6, 7].

The best way to prevent it is to eat more carefully, choosing food not only helps prevent intestinal perforation due to foreign objects but also avoids other problems such as reducing constipation or intestinal obstruction due to bezoar. Treatment of intestinal perforation due to fish bones will be different. The mildest case can be treated conservatively; however, in most cases, surgery is needed to resect the appendix, treat peritonitis and unhealed damage to other organs. Preoperative ultrasound and CT assessment would be useful as a guide to help the surgeon remove the foreign body in the same setting as the appendectomy [8, 9].

In conclusion, although it is not uncommon for ingested foreign bodies to pass through the digestive tract, it should be noted that appendicitis is not always simple. Fish bones are sometimes difficult to see on imaging, requiring the radiologist to actively look for foreign bodies on the images to ensure the diagnosis. At the same time, the surgeon must find and remove the fish bone, otherwise the fish bone can become a source of infection in the abdomen. Furthermore, the surgeon needs to actively evaluate and manage other related complications that may occur such as intestinal perforation in other segments, in addition to performing an appendectomy. This case highlights the importance of early recognition and appropriate management of perforated appendicitis due to foreign body.

## Conflict of interest statement

None declared.

## Funding

None declared.

## Author contributions

The authors contributed equally.

## Data availability

All available data were included in this published article.

## Consent statement

Informed consent has been obtained from the patient.
